# Effect of Neuromuscular Blockade on Intraoperative Respiratory Mechanics and Surgical Space Conditions during Robot-Assisted Radical Prostatectomy: A Prospective Randomized Controlled Trial

**DOI:** 10.3390/jcm10215090

**Published:** 2021-10-29

**Authors:** Chang-Hoon Koo, Insun Park, Sungmin Ahn, Sangchul Lee, Jung-Hee Ryu

**Affiliations:** 1 Department of Anesthesiology and Pain Medicine, Seoul National University Bundang Hospital, Seongnam 13620, Korea; vollock9@gmail.com (C.-H.K.); pis121@hanmail.net (I.P.); spikp50@gmail.com (S.A.); 2 Department of Anesthesiology and Pain Medicine, College of Medicine, Seoul National University, Seoul 03080, Korea; 3 Department of Urology, Seoul National University Bundang Hospital, Seongnam 13620, Korea

**Keywords:** neuromuscular blockade, radical prostatectomy, laparoscopy, robot-assisted surgery

## Abstract

The aim of this study was to investigate whether deep neuromuscular blockade (NMB) may affect intraoperative respiratory mechanics, surgical condition, and recovery profiles in patients undergoing robot-assisted radical prostatectomy (RARP). Patients were randomly assigned to the moderate or deep NMB groups. Pneumoperitoneum was maintained with carbon dioxide (CO_2_) insufflation at 15 mmHg during surgery. The primary outcome was peak inspiratory pressure (PIP) after CO_2_ insufflation. Mean airway pressure (P_mean_) and dynamic lung compliance (C_dyn_) were also recorded. The surgeon rated the surgical condition and surgical difficulty on a five-point scale (1 = extremely poor; 2 = poor; 3 = acceptable; 4 = good; 5 = optimal). Recovery profiles, such as pulmonary complications, pain scores, and recovery time, were recorded. We included 58 patients in this study. No significant differences were observed regarding intraoperative respiratory mechanics including PIP, P_mean_ and C_dyn_, between the two groups. The number of patients with optimal surgical conditions was significantly higher in the deep than in the moderate NMB group (29 vs. 20, *p* = 0.014). We found no differences in recovery profiles. In conclusion, deep NMB had no significant effect on the intraoperative respiratory mechanics but resulted in optimal endoscopic surgical conditions during RARP compared with moderate NMB.

## 1. Introduction

Robot-assisted laparoscopic radical prostatectomy (RARP) is a representative robot-assisted surgery usinghigh-pressure carbon dioxide (CO_2_) for insufflation into the abdominal cavity, to ensure the visibility of the operative field and to protect other organs. To perform RARP using a transperitoneal approach, it is necessary to maintain a steep Trendelenburg position to expose the bladder and the prostate. This surgical position moves the diaphragm toward the head, and may cause excessive peak inspiratory pressure (PIP) and barotrauma in the lung parenchyma in patients on mechanical ventilation [1]. Overall, respiratory mechanics may be impaired, such as a decrease in functional residual capacity and lung compliance [2]. Respiratory impairments can persist for up to 24 h after surgery, delaying patient recovery [3].

The risk of prostate cancer increases with age, especially in men who are 65 years of age or older, with an incidence rate of 1 in 8 men [4]. Hence, RARP is often performed on the elderly, who may have deteriorated respiratory mechanics, as aging decreases lung compliance, increases closing volume, and aggravates pulmonary complications [5]. Therefore, careful management is necessary among elderly patients undergoing RARP, especially those with respiratory problems. Further more, due to the narrow pelvic space and limited exposure of the apical portion of the prostate, RARP procedures, such as dorsal vein complex ligation, nerve sparing, and vesicourethral anastomosis are difficult and complex [6]. Therefore, securing a sufficient visual field may contribute to better surgical outcomes.

During general anesthesia, neuromuscular blockade (NMB) is essential for tracheal intubation and optimal surgical conditions. Several studies have evaluated the effect of NMB on surgical conditions and demonstrated that deep NMB improves conditions more than moderate NMB [7,8]. It has also been shown that deep NMB, along with the use of sugammadex, improves the surgical conditions of radical prostatectomies [9]; this can be particularly beneficial in laparoscopic surgery performed in a narrow retroperitoneal space. Deep NMB has been widely used for RARP, but its efficacy in improving respiratory mechanics among elderly patients undergoing RARP remains unclear. A better understanding of this efficacy may have implications for some common surgical procedures and lines of surgery management or treatment. Therefore, we compared the respiratory mechanics, surgical conditions, and recovery profiles between moderate NMB and deep NMB in patients undergoing RARP.

## 2. Materials and Methods

### 2.1. Study Design

This was a prospective, randomized controlled trial. The protocol was approved by the Institutional Review Board of Seoul National University Bundang Hospital (B-1910-572-007) and registered at ClinicalTrials.gov (NCT 04174222). This study was performed from November 2019 to August 2020 at Seoul National University Bundang Hospital. Written informed consent was obtained from all patients before surgery.

### 2.2. Participants

Patients over 19 years of age with a physical status of I or II according to the American Society of Anesthesiology, and scheduled for elective RARP under general anesthesia, were included in this study. The exclusion criteria were, a history of neuromuscular disorders impairing neuromuscular function, renal insufficiency, moderate to severe obstructive/restrictive pulmonary disease, a body mass index over 30 kg/m^2^, a history of NMB agent allergy, or scheduled for transfer to the intensive care unit.

### 2.3. Randomization and Intervention

Eligible patients were randomly assigned to either the moderate NMB group (n = 29) or deep NMB group (n = 29) using computer-generated randomization numbers (Random Allocation Software version 2.0, Isfahan University of Medical Sciences, Isfahan, Iran), which were sealed in an opaque envelope. Randomization was conducted by a nurse who was not involved in this study. The patients, the surgeon who rated the surgical conditions, and the outcome assessors were blinded to the group assignments. In the moderate NMB group, a train-of-four (TOF) count of 1 to 2 was maintained during surgery and 2 mg/kg of sugammadex was administered for the reversal of NMB after surgery. In the deep NMB group, a post-tetanic count (PTC) of 1 to 2 was maintained during surgery and 4 mg/kg of sugammadex was used for reversal. Appendix A summarizes the details of both TOF count and PTC. A single experienced urologist performed the RARP on all patients. During surgery, pneumoperitoneum was maintained with CO_2_ insufflation at 15 mmHg. The surgeon was allowed to increase the pressure to 20 mmHg, if needed.

### 2.4. Randomization and Intervention

Premedication with intravenous midazolam (0.02 mg/kg) was performed administered in the reception area. In the operating room, routine monitoring, including noninvasive arterial pressure measurements, electrocardiography, and pulse oximetry, were appliedwas conducted. In addition, acceleromyography (TOF-WatchSX, MSD BV, Oss, The Netherlands) was carried out at the adductor pollicis muscle to monitor the level of NMB. Anesthesia was induced with propofol (1~2 mg/kg) and remifentanil (3.0 ng/mL of effect site concentration). After loss of consciousness, the TOF-WatchSX was calibrated and stabilized, a 50 Hz tetanic stimulation was applied for 5 s, and a series of TOF measurements were documented for 1 min until a stable baseline was obtained (<5% variation Subsequently, rocuronium (0.6 mg/kg) was administered intravenously, and patients were intubated with a plain endotracheal tube (Covidien, Mansfield, MA, USA) with an inner diameter of 7.5 mm, using a direct or video (AceScope, Ace Medical, Seoul, Korea) laryngoscope. After intubation, the lungs were mechanically ventilated (Datex-Ohmeda, GE Healthcare, Chicago, IL, USA) with an inspired oxygen fraction of 0.5 with a fresh gas flow at 2 L/min, a tidal volume of 6 mL/kg (ideal body weight), a positive end expiratory pressure of 5 cmH_2_O, and a respiratory rate of 16 breaths/min. The radial artery was cannulated with a 20-gauge catheter to monitor arterial blood pressure and perform arterial blood gas analysis. Anesthesia was maintained with 7.0 to 8.0 vol% desflurane in oxygen–air (50%/50%) and 2.0 to 3.0 ng/mL of remifentanil. A bolus dose of intravenous rocuronium (5–10 mg) was used to maintain the TOF count of 1–2 and PTC of 1–2, in the moderate and deep NMB groups, respectively. TOF and PTC were measured every 30 s and 5 min, respectively. During RARP, both hands and digits weare free, allowing us to use acceleromyography. At the end of the surgery, NMB was reversed using 2 mg/kg (moderate NMB group) or 4mg/kg (deep NMB group) of sugammadex. Patients were extubated at TOF > 0.9 and transferred to the post anesthesia care unit (PACU).

### 2.5. Surgery

All surgeries were performed by a single experienced surgeon at our institution. RARP (with or without pelvic lymph node dissection) was performed via a transperitoneal approach by use of the 4-armed da Vinci sSurgical rRobot sSystem based on the physician’s and patient’s discretion. The neurovascular bundle was spared regardless of preoperative erectile function unless this procedure violated oncological principles [10].

### 2.6. Measurements

During surgery, an investigator recorded the peak inspiratory pressure (PIP) and mean airway pressure (Pmean), which were measured through a ventilator at six time points: (1) immediately after tracheal intubation (T1); (2) immediately after CO_2_ insufflation (T2); (3) 30 min after CO_2_ insufflation (T3); (4) 60 min after CO_2_ insufflation (T4); (5) immediately after CO_2_ removal (T5); and (6) at the end of surgery (T6). The dynamic lung compliance (Cdyn) was calculated using the following formula: Tidal volume/(PIP − PEEP). Arterial blood gas analyses were conducted at pre-defined time -points to measure the arterial partial pressure of oxygen (PaO_2_) and arterial partial pressure of carbon dioxide (PaCO_2_). At the end of surgery, endoscopic surgical conditions and surgical difficulty were evaluated by the operator using a 5-point scale (1 = extremely poor; 2 = poor; 3 = acceptable; 4 = good; 5 = optimal) [8]. During the PACU stay, all patients were assessed every 15 min, using the modified Aldrete scores, until discharge. An independent nurse performed arterial blood gas analysis after 30 min after PACU admission. Desaturation (SpO_2_ < 8/min), pain scores, number of rescue analgesic requirements, and postoperative nausea and vomiting (PONV) were recorded. All patients underwent chest radiography on postoperative day 1. The primary outcome was PIP after CO_2_ insufflation. Secondary outcomes were P_mean_, C_dyn_, PaO_2_, PaCO_2_, endoscopic surgical conditions, and recovery profiles including postoperative pulmonary complications, pain scores, analgesic requirements during PACU stay, PONV, and recovery time (a modified Aldrete score ≥ 9, duration of PACU stay, and length of hospital stay (LOS)).

### 2.7. Sample Size Calculation

The sample size was calculated based on the data obtained from the previous study [11]. The PIP was 28.3 ± 4.1 mmHg at 30 min after CO_2_ insufflation during RARP in the moderate NMB group. The Ddecrease inof PIP to 25 mmHg by in the deep NMB group was considered to be clinically significant. Twenty- nine patients per group were calculated included in the calculations (α = 0.05, power = 0.8), considering the 10% dropout rate.

### 2.8. Statistical Analysis

The iIndependent t-test and the Mann-Whitney U tests were performed for the comparison of continuous data between the moderate and deep NMB groups according to the results of the Shapiro-Wilk normality test. The Chisquare test or Fisher’s exact test were was used for comparisons of categorical data according to the expected frequency in each cell of the table. Two sided *p*-value < 0.05 was considered statistically significant. All statistical analyses were performed via SPSS software, version 25 (IBM, Chicago, IL, USA).

## 3. Results

**Figure 1:**
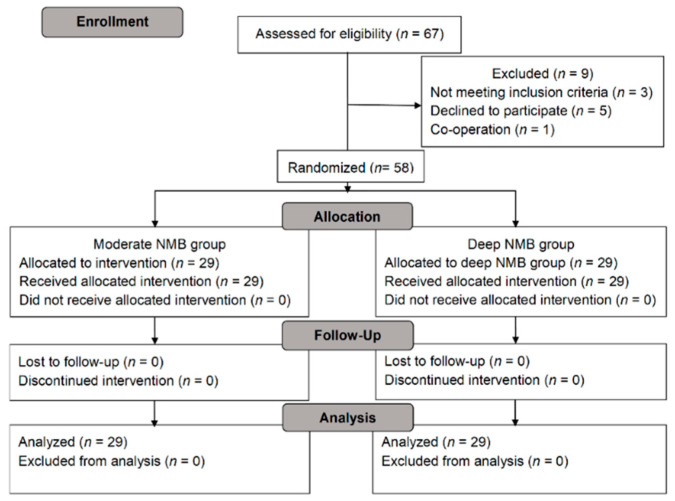
Consort flow diagram of the patients.

Of the 67 patients who underwent RARP, nine patients were excluded (5five: refused to participate; three3: exclusion criteria; one1: co-operation); hence, a total of 58 patients were analyzed in this study. Among these patients, 29 patients were equally allocated to both the moderate NMB group and the deep NMB group, respectively (Figure 1 and Table 1).

### 3.1. Respiratory Mechanics

**Figure 2:**
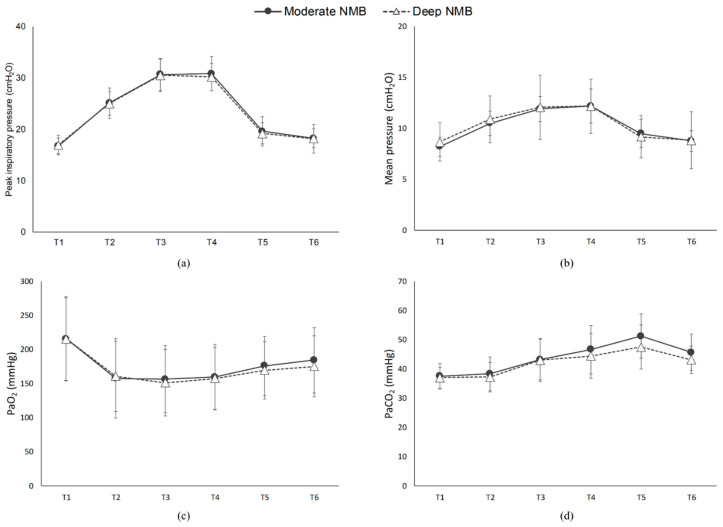
The effects of moderate or deep neuromuscular blockade were assessed during robot-assisted radical prostatectomy(RARP); (1) immediately after tracheal intubation (T1); (2) immediately after CO_2_ insufflation (T2); (3) 30 min after CO_2_ insufflation (T3); (4) 60 min after CO_2_ insufflation (T4); (5) immediately after CO_2_ removal (T5); (6) at the end of surgery (T6). (Moderate [●]: 1–2 twitch in the train-of-four stimulation; deep [△]: 1–2 twitches in the post-tetanic count.) (**a**) Peak inspiratory pressure, (**b**) mean airway pressure, (**c**) partial pressure of arterial oxygen, (**d**) and partial pressure of arterial carbon dioxide at different surgical stages in general anesthetized patients undergoing RARP.

The change in PIP during surgery is shown in Figure 2a. The baseline values of PIP (T1) were similar between the two groups. As expected, CO_2_ insufflation (T2) and Trendelenburg position (a 30 degree incline with the feet elevated above the head) (T3) significantly increased PIP in both groups, respectively (*p* < 0.001). However, repeatedmeasuresd ANOVA revealed that no effect of the interaction between time and group on the PIP was found (*p* = 0.701). Similarly, no significant differences in Pmean were not observed between the two groups at anyall time -points (Figure 2b). When comparing patients in the moderate NMB group versus to the deep NMB group, the Cdyn at T3 and T4 were was 15 ± 2.3 vs. 15.2 ± 2.0 mL/cmH_2_O, and 15 ± 2.3 vs. 15.4 ± 1.7 mL/cmH_2_O, with *p* = 0.713 and 0.443, respectively. In terms of oxygenation, the values of PaO_2_ and PaCO_2_ were comparable between the two groups during RARP (Figure 2c,d).

### 3.2. Endoscopic Surgical Conditions and Surgical Difficulty

The deep NMB group showed a significantly higher number of patients who had optimal conditions during RARP compared to the moderate NMB group (29 vs. 20, *p* = 0.014, Table 2). Meanwhile, no significant difference was noted in surgical difficulty between the deep NMB group and the moderate NMB group (*p* = 0.823, Table 2). The number and duration of increase in CO_2_ insufflation pressure were comparable between the two groups (*p*s ≥ 0.05, Table 2).

### 3.3. Recovery Profiles

There wae no significant difference between the two groups in terms of recovery profile including postoperative pulmonary complications, pain scores, the number of analgesic requirements, the incidence of PONV, and recovery time (*p*s ≥ 0.05, Table 3).

## 4. Discussion

This study revealed that deep NMB had no significant effect on the PIP, P_mean_, C_dyn_, or oxygenation. However, the number of optimal surgical conditions was significantly higher in the deep NMB group than in the moderate NMB group, with a similar level of surgical difficulty in both groups. Moreover, no significant differences in recovery profiles were noted between the two groups.

Hence, contrary to the expectation that NMB may have the potential to alter the viscoelastic mechanical properties of the chest wall by reducing chest wall rigidity via the relaxation of skeletal muscles, no definitive impact of NMB on respiratory mechanics was found. These results are consistent with those of a previous study [12] which described the effect of different levels of NMB on respiratory mechanics during robotic surgery. The authors demonstrated that changes in the level of NMB did not contribute to alterations in chest wall pressure. In terms of abdominal elastic properties, our findings are supported by a previous animal study that compared PIP and intra-abdominal pressure (IAP) during CO_2_ insufflation in a pig model [13]. The authors reported that NMB could not decrease PIP or IAP in pigs undergoing laparoscopy and explained that the same volume of CO_2_ in the abdominal cavity produced similar IAP, causing comparable PIP. Given the limited number of human trials, it is noteworthy that this is a clinical study reporting the effect of the depth of NMB on PIP among patients undergoing RARP.

Consistent with previous studies, the surgical condition was considerably better in the deep NMB group than in the moderate NMB group. It is interesting to note that all patients in the deep NMB group processed optimal surgical conditions regardless of the surgical difficulty. This finding correlates fairly well with previous result [14]. Dubois et al. [14] verified the hypothesis that deep NMB significantly improved surgical conditions in patients undergoing laparoscopic hysterectomy. They found that deep NMB always guaranteed excellent pelvic laparoscopic conditions. This result was explained by the prevention of abdominal wall muscle contractions during surgery. For many years, deep NMB has been considered a promising approach to produce better surgical space conditions. A recent meta-analysis noted that deep NMB improved surgical space conditions more than moderate NMB [15]. However, most previous works have focused on surgeries with low pneumoperitoneum pressure (8–13 mmHg). Moreover, most studies have focused on intraperitoneal surgeries, such as laparoscopic cholecystectomy, bariatric surgery, or colorectal surgery. To the best of our knowledge, there are only two studies comparing surgical space conditions between deep and moderate NMB in patients undergoing retroperitoneal laparoscopic surgery [8,16]. Martini et al. [8] kept the IAP constant at 9–11 mmHg, and found that the mean surgical rating was significantly higher in the deep NMB group than in the moderate NMB group (4.7 vs. 4.0). Yoo et al. [16] decreased the IAP to 8 mmHg and concluded that deep NMB better facilitated the surgical procedures at low IAP than moderate NMB. We used IAP from 15 to 20 mmHg, and hence, provide evidence that deep NMB improves surgical space conditions under high IAP [15]. A recent study comparing the effects of deep and moderate neuromuscular block on respiratory system compliance and surgical space conditions during RARP was similar in design to our study; however, the IAP in the previous paper was fixed at 10mmHg [17]. Our patients underwent RARP at a higher IAP (15mmHg), and the surgeon was allowed to increase the IAP up to 20mmHg. Therefore, there was a significant difference in the surgical space condition in this paper, which did not differ between the two groups in the previous paper [17]. We hope that this study is the first step toward enhancing our understanding of deep NMB in retroperitoneal laparoscopic surgery performed under high IAP.

The deep NMB group showed no significant difference in recovery profiles in our study; postoperative pulmonary complications, including desaturation, respiratory depression, and atelectasis, were comparable between the two groups, which may be explained by sugammadex administration to both groups. This is in agreement with a previous study that compared results between deep and moderate NMB, in patients undergoing transurethral resection of the bladder [18], and found that no postoperative pulmonary complications occurred in either cases.

Although several studies showed that deep NMB causes a significant reduction in early postoperative pain [16,19], our results differ. Our results may be attributed to the controlled IAP we maintained, whereas, most previous studies changed the IAP, and reported that deep NMB could achieve a sufficient surgical view, despite a lower IAP. Decreasing the IAP is known to reduce postoperative pain [20]. Since we maintained a high IAP at 15 mmHg constantly during RARP, most patients in both the groups had similar high pain scores (numerical rating scale ≥ 6).

This study had a few limitations. First, deep NMB was achieved by the intermittent administration of rocuronium rather than continuous infusion. This was because fluid restriction is recommended to optimize the surgical field and to minimize postoperative complications during RARP [21]. Continuous infusion may limit the NMB effect of rocuronium. In addition, a previous study demonstrated that the surgical condition was comparable between continuous rocuronium infusion and a bolus of rocuronium [22]. Second, the urologist evaluated the surgical space conditions and surgical difficulties as soon as at the surgery ended. To enhance the objectivity of surgical view scores and surgical difficulties, more assessors may participate in the evaluation of the surgical space conditions and difficulties with video images. Third, this study included non-obese patients. However, since obesity is known to be a risk factor for impaired respiratory mechanics [23,24] and complications [25], further study is needed to explore the effect of NMB on intraoperative respiratory mechanics in obese patients.

## 5. Conclusions

In conclusion, the evidence from this study shows that deep NMB has no effect on respiratory mechanics, but may enhance the surgical space conditions for patients undergoing RARP, under high IAP. Further study with a variety of laparoscopic surgeries is needed on the association between of deep NMB and respiratory profiles with a variety of laparoscopic surgeries is needed.

## Figures and Tables

**Table 1:** Characteristics of patients, surgery and anesthesia.

	Moderate NMB (*n* = 29)	Deep NMB (*n* = 29)	*p*-Value
Age, years	66.9 (6.7)	65.9 (5.7)	0.543
Weight, kg	71.2 (9.0)	69.6 (7.3)	0.458
Height, cm	167.1 (5.1)	168.1 (5.4)	0.474
BMI, kg/m^2^	25.4 (2.5)	24.4 (2.8)	0.156
ASA physical status (I/II), n (%)	6 (20.7)/23 (79.3)	5 (17.2)/24 (82.8)	0.738
Pulmonary function test, n (%)			0.254
Normal	28 (96.6)	26 (89.7)	
Borderline obstructive	0	2 (6.9)	
Mild obstructive	0	1 (3.4)	
Mild restrictive	1 (3.4)	0	
Duration of surgery, min	147.8 (25.6)	149.1 (23.0)	0.830
Duration of anesthesia, min	175.0 (25.0)	180.0 (32.5)	0.658
Duration of CO_2_ insufflation, min	118.1 (22.6)	120.9 (23.8)	0.652
Crystalloid, mL	655.5 (150.9)	701.7 (211.9)	0.343
Estimated blood loss, mL	131.0 (98.4)	123.5 (67.5)	0.733
Rocuronium, mg	68.1 (14.1)	107.5 (21.5)	<0.001

Data are presented as mean (standard deviation) or number of patients (%). Abbreviations: NMB, neuromuscular blockade; BMI, body mass index; ASA, American Society of Anesthesiologists.

**Table 2:** Surgical conditions and difficulty.

	Moderate NMB (*n* = 29)	Deep NMB (*n* = 29)	*p* Value
Endoscopic surgical conditions, n (%)			0.014
Extremely poor	0	0	
Poor	1 (3.4)	0	
Acceptable	2 (6.9)	0	
Good	6 (20.7)	0	
Optimal	20 (69.0)	29 (100)	
Surgical difficulty, n (%)			0.823
Extremely poor	3 (10.3)	3 (10.3)	
Poor	14 (48.3)	12 (41.4)	
Acceptable	8 (27.6)	10 (34.5)	
Good	3 (10.3)	4 (13.8)	
Optimal	1 (3.4)	0	
Increase in CO_2_ insufflation pressure			
Number	1 (1–2)	1 (1–2)	0.906
Duration, min	10 (5–27.5)	12.5 (5–20)	0.809

Data are presented as median (interquartile range) or number of patients (%). Abbreviations: NMB, neuromuscular blockade.

**Table 3:** Postoperative complications.

	Moderate NMB (*n* = 29)	Deep NMB (*n* = 29)	*p* Value
SpO_2_ < 90% or RR < 8/min, n (%)	2 (6.9)	1 (3.4)	0.553
Lowest SpO_2_, %	95.7 (2.7)	96.1 (1.9)	0.504
Atelectasis, n (%)	2 (6.9)	0	0.150
PaO_2_, mmHg	89.5 (20.6)	91.6 (24.7)	0.735
PaCO_2_, mmHg	34.6 (6.9)	35.5 (6.9)	0.637
Pain score, n (%)			0.106
NRS 3	1 (3.4)	0	
NRS 6	16 (55.2)	8 (27.6)	
NRS 7	7 (24.1)	12 (41.4)	
NRS 8	5 (17.2)	9 (31.0)	
Analgesic requirement	2 (1.5–3)	2 (2–3)	0.413
PONV, n (%)	1 (3.4)	0	0.313
a modified Aldrete score ≥ 9, min	15 (6.0)	15 (11.5)	0.826
PACU stay, min	50 (14)	50 (13)	0.950
Length of hospital stay, day	8 (1)	8 (2)	0.616

Data are presented as mean (standard deviation), median (interquartile range) or number of patients (%). Abbreviations: NMB, neuromuscular blockade; SpO_2_, saturation of percutaneous oxygen; RR, respiratory rate; PaO_2_, partial pressure of arterial oxygen; PaCO_2_, partial pressure of carbon dioxide; NRS, numerical rating scale; PACU, post-anesthesia care unit.

## Data Availability

The datasets generated and analyzed during this study are available on request to the corresponding author on reasonable request.

## Appendix A

We summarized the meaning of what a train-of- four (TOF) count and a post-tetanic count (PTC) mean according to the previous literatures [26,27]. Both TOF and PTC are indicators of the depth of neuromuscular blockade (NMB). During general anesthesia, the degree of NMB can be monitored by evoked muscle responses. Consecutive fFour consecutive electrical stimulations are delivered through motor nerves, and the number of muscle twitches (range 0–4) are observed and recorded. At this time, the number of muscle twitches is called a TOF count. For example, a TOF of 3three means that there are 3three muscle twitches after electrical stimulation. Rocuronium administration decreases the TOF count. If a large dose of rocuronium is administered, the TOF count will be zero. When there are no responses to four consecutive stimuli, PTC is useful to distinguish between an intense block and a deep block. A strong stimulus (50 Hz for 5 s) promotes acetylcholine synthesis and mobilization at the nerve terminal. These This, socalled, tetanic stimuli enable the muscle response to subsequent single twitch stimulation. At this time, the number of muscle responses to single twitch stimulation after tetanic stimulus is called PTC. During an intense block, there is no response to post-tetanic stimulation. A response to post-tetanic stimulation means deep NMB.
